# Health-related quality of life became worse in short-term during treatment in head and neck cancer patients: a prospective study

**DOI:** 10.1186/s12955-020-01543-5

**Published:** 2020-09-16

**Authors:** Emanuelle do Nascimento Santos Lima, Isabela Borges Ferreira, Paula Philbert Lajolo, Carlos Eduardo Paiva, Yara Cristina de Paiva Maia, Geórgia das Graças Pena

**Affiliations:** 1 Graduate Program in Health Sciences, Federal University of Uberlandia, Pará Av, 1720 / 2U, Campus Umuarama, Uberlandia, Minas Gerais 38400-902 Brazil; 2 Department of Clinical Oncology, Clinical Hospital, Federal University of Uberlandia, Pará Av, 1720 / sala 9, Campus Umuarama, Uberlandia, Minas Gerais 38.405-320 Brazil; 3 Department of Clinical Oncology and Research Group on Palliative Care and Health-Related Quality of Life (GPQual), Barretos Cancer Hospital, Barretos, Antenor Duarte Viléla St, 1331, Dr. Paulo Prata, Barretos, SP 14784-400 Brazil; 4 School of Medicine; Nutrition Course, Federal University of Uberlandia, Pará Av, 1720 / 2U, Campus Umuarama, Uberlandia, Minas Gerais 38400-902 Brazil

**Keywords:** Head and neck neoplasms, Health-related quality of life, Radiotherapy, Chemotherapy

## Abstract

**Background:**

Quality of life (QoL) is influenced in head and neck cancer (HNC) patients by a set of factors related to diagnosis, treatment and tumor impacts. The aim of this study was to evaluate the Quality of Life (QoL) changes in Head and Neck cancer (HNC) patients during treatment (radiotherapy and/or chemoradiotherapy).

**Methods:**

QoL was evaluated prospectively in 63 HNC patients during radiotherapy and/or chemoradiotherapy at three moments: before or at beginning (T0), in the middle (T1 ~ four weeks) and immediately at the end (T2 ~ eight weeks) of treatment. The differences between the scores at different time points was verified using Friedman’s non-parametric test. Negative changes between time points were evaluated, with differences (delta) of ±10 points being considered to be clinically significant.

**Results:**

The total mean age was 59.1 ± 9.5y, and 82.5% were male. The oral cavity and larynx were more frequent tumors. The functional score for ‘role’ was decreased at time points T1 and T2 as compared to T0, while an improvement in scores was observed for cognitive function. Several physical symptoms also worsened over time, such as: fatigue, nausea and vomiting, dry mouth and sticky saliva, swallowing and skin symptoms, senses and teeth problems. A high frequency of altered and clinically meaningful values were observed for most of domains, ranging from 6 to 74%.

**Conclusions:**

The QoL became worse at approximately one month after treatment beginning in HNC patients, and this remained until the end of therapy. Protocols directing to early nutritional counseling and management of symptoms of nutritional impact are important to improve clinical outcomes. This is part of preventive actions aiming to make the exhausting treatment process less traumatic and easier to complete.

## Background

Head and neck cancer (HNC) comprises tumors that affect the larynx, pharynx and oral cavity [1]. It is the sixth most prevalent type of cancer in the world [2] and was responsible for 22,200 (salivary gland cancer) to 177,400 (lip and oral cavity cancer) deaths in 2018 [3].

Quality of life (QoL) for HNC patients is influenced by diagnosis, tumor impact and several side effects arising from different types of treatment [2, 4]. The head and neck is an anatomical region with many essential structures for swallowing, feeding, speech and breath. In HNC patients these functions can be affected, leading to losses in aspects related to social interaction and functionality [5] and resulting in physical deterioration, financial burden and a low commitment to treatment. In addition, tumor site and need for surgical resections can lead to changes in appearance, body image and emotional impacts [2]. This clinical scenario is accentuated by the presence of symptoms like xerostomia, dysgeusia, oral mucositis, pain and dysphagia [6, 7], probably as a treatment result. This set of changes negatively impacts health-related quality of life (HRQOL) of these patients [8].

HRQOL can be defined as the subjective perception of individual concerning how the diseases and therapies affect the various domains of their life, such as psycho-social and physical domains [9–11]. In this sense, the maintenance and improvement of QoL should be considered one of the treatment goals [2, 12]. Thus, to analyze these scales should allow one to evaluate all aspects related to patient’s health in an integrated manner.

Although an impact on HRQOL is expected in HNC patients, knowledge of when this impact starts and begins to increase is important. Such evidence enables early intervention and health counselling; in addition to providing adequate support at the time needed for patients and their families, this also supports the continuity of treatment and avoids and/or minimizes long-term impacts.

Most of studies on HRQOL in HNC patients have investigated different time points, for example three months, six months and a year after treatment [13–16]. Thus, studies evaluating this context in short-term are still scarce. We hypothesized that HRQoL would become worse in short-term during treatment. Thus, the aim of this study was to evaluate the HRQOL in HNC patients before or at beginning (T0), in the middle (T1 ~ four weeks) and immediately at the end (T2 ~ eight weeks) of treatment.

## Methods

### Participants, design of study and ethical aspects

A prospective study was carried out from July 2017 to November 2018 with HNC patients at a tertiary Brazilian hospital. These patients were evaluated at three time points: before or at beginning (T0), in the middle (T1 ~ four weeks) and immediately at the end (T2 ~ eight weeks) of antineoplastic treatment. The time of the evaluations for each individual varied from approximately seven to eight weeks according to the treatment protocol used.

This study was approved by Human Research Ethics Committee of Federal University of Uberlandia (protocol number 65340116.8.0000.5152) and in accordance with of the Helsinki Declaration. All participants signed to provide written informed consent before being considered for the study.

The study included all patients with primary HNC, undergoing radiotherapy and/or chemoradiotherapy, with or without surgery, independent of tumor stage (advanced or initial) and aged 18 years or over. Patients were considered to be at T0 when they had not begun any treatment or if they had received up to seven initial sessions of radiotherapy, since these patients did not report collateral effects. Patients with distant metastasis at time T0 or who were submitted to radiotherapy and chemotherapy for other types of cancer in the last 10 years were excluded.

In order to know if sample was large enough to test required outcomes, a post hoc test was performed using G* Power software, version 3.1. Using the Wilcoxon signed-rank, two-tailed test, with an error of 0.07 and a sample size of 63, a power of 0.97 was obtained. Therefore, the sample has enough power for the analyses in the present study.

### Procedures

At the three time points, patients were invited to respond questions about sociodemographic, economic, clinical and anthropometric aspects and these were related to treatment data. Additional treatment data were collected from clinical records, such as Tumor Node Metastasis (TNM) Classification of Malignant Tumors by International Union for Cancer Control (UICC) and 7^th^ edition of the American Joint Committee on Cancer (AJCC) [17]. Cancer treatments were planned in a multidisciplinary manner, taking into account the type of tumor and staging, in addition to the individual characteristics of each patient, and followed the institutional protocol for cancer treatment.

### Instruments

HRQOL was evaluated using the questionnaires developed by European Organization for Research and Treatment of Cancer (EORTC), in the Brazilian Portuguese versions, after gaining permission. Three questionnaires were applied at the time points (T0, T1 and T2): the Core Questionnaire (EORTC QLQ-C30), the disease-specific HNC module (EORTC QLQ-H&N35) and the new revised module for HNC (EORTC QLQ-H&N43).

The EORTC QLQ-C30 [18] contains 30 questions and addresses general aspects of QoL in patients with cancer, with 28 questions assessed on a 4-point Likert scale and two questions assessed on a 7-point linear scale. This questionnaire considers five functional scales (physical, role, emotional, cognitive and social), three symptom scales (fatigue, nausea and vomiting and pain), six items (dyspnea, insomnia, appetite loss, constipation, diarrhea and financial difficulties) and one global scale. Higher scores on functional and global scales indicate better QoL. However, for scales and items related to symptoms higher scores indicate worsened QoL.

EORTC QLQ-H&N 35 [19] and EORTC QLQ-H&N 43 [8] questionnaires contain 35 questions with 18 scales and 43 questions with 19 scales, respectively. In both questionnaires, all questions are assessed on a 4-point Likert scale, where higher scores indicate a worse QoL. The two questionnaires are specific for HNC patients and evaluate symptoms, sexuality and body image. However, despite presenting similar questions, the QLQ-H&N 43 contains additional questions, addressing skin, neurological and shoulder problems, while other questions were removed from the traditional version, such as nutritional supplements and use of a feeding tube [8]. The values obtained from scales were converted into scores from 0 to 100, according to the recommendations of EORTC [20].

The differences between the points during follow-up can be clinically relevant. There is a clinical significance concept that is very important in practice. According to Sloan et al. (2006) since a specific population cutoff point, even some finding that does not have a statistically significant *p*-value, they could be clinically significant. In this sense, we calculated the difference between the scores by time points, achieving our specific cutoff point, i.e., a ∆ (delta) for T1-T0, T2-T0 and T2-T1. The specific value for our population was similar and defined by following calculation: effect size (0.5) x Standard Deviation of study population (SD = 21.12) = 10.56 [21]. So, our cut-off was considered ±10 points. Any scales with this delta ≥10 or ≤ − 10 points was considered as clinically relevant, similar to a previous study [8]. In our study, only the worsening between times was shown, i.e., the clinical negative significant changes, respecting the negative direction of the scales and items.

Data referring to nutritional status of patients were obtained from Patient-Generated Subjective Global Assessment (PG-SGA) applied at the three time points of the study.

### Statistical analyses

Firstly, the Kolmogorov-Smirnov normality test was performed. Descriptive statistics were used to describe clinical data and QoL scores, and expressed as percentage, mean and standard deviations or median and interquartile range (IQ25–75%). Differences between scores at the three time points of study were estimated by Friedman’s non-parametric test and Dunn post hoc test, adjusted by Bonferroni.

Statistical analyses were performed using Statistical Package Social Sciences (SPSS Statistics for Windows, version 20.1, SPSS®, Inc., Chicago, USA) considering a *p*-value ≤0.05 and 95% confidence interval.

## Results

**Fig. 1:**
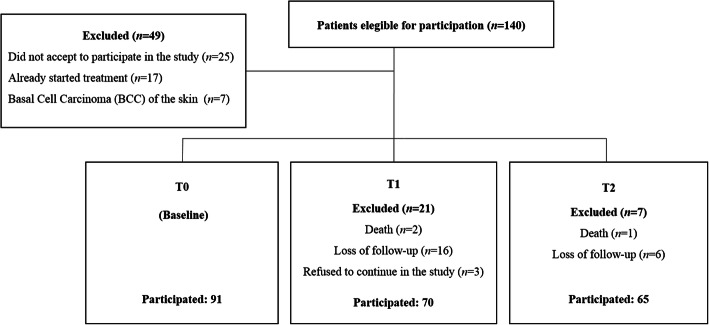
Diagram reporting the number of head and neck cancer patients screened and recruited in this study

During the study period, 140 patients were approached; 25 declined to participate, and 24 did not meet the inclusion criteria, resulting in 91 patients at baseline and 63 at the end of study with complete longitudinal information (Fig. 1). Of the 63 patients, 82.5% were male, with a mean age of 59.1 ± 9.5 years. Cancer of the larynx, oral cavity and pharynx were the more frequent tumor types. The majority of patients were in advanced (T3-T4; 57.1%) stage cancer and chemoradiotherapy and isolated radiotherapy were the treatments most frequently prescribed. Regarding nutritional diagnosis by PG-SGA, 47.6% of patients were deemed to have moderate malnutrition (Table 1).

**Table 1:** Clinical data of patients with head and neck cancer

Variables	Total sample (***n*** = 91)	Analized sample (***n*** = 63)
	n (%)	
**Sex,** Male	70 (76.9)	52 (82.5)
**Age** (years), mean (SD)	60.6 (10.9)	59.1 (9.5)
**Tumor site**		
Oral cavity (tongue, mouth floor and lip)	30 (33.0)	21 (33.3)
Nasal cavity	4 (4.4)	2 (3.2)
Larynx	32 (35.2)	22 (34.9)
Pharynx (hypopharynx, oropharynx and nasopharynx)	22 (24.2)	17 (27.0)
Others (jaw, cervical and parathyroid)	3 (3.3)	1 (1.6)
**Tumor T stage**		
I	10 (11.0)	10 (15.9)
II	19 (20.9)	15 (23.8)
III	32 (35.2)	23 (36.5)
IV	23 (25.3)	13 (20.6)
X	5 (5.5)	2 (3.2)
Not specified or unknown	2 (2.2)	–
**Tumor N stage**		
0	37 (40.7)	32 (50.8)
I	17 (18.7)	11 (17.5)
II	20 (22.0)	13 (20.7)
III	8 (8.8)	4 (6.3)
X	6 (6.6)	2 (3.2)
Not specified or unknown	3 (3.3)	1 (1.6)
**Tumor M stage**		
0	55 (60.4)	40 (63.5)
1	10 (11.0)	7 (11.1)
X	21 (23.1)	15 (23.8)
Not specified or unknown	5 (5.5)	1 (1.6)
**Treatment**		
Radiotherapy	21 (23.1)	16 (25.4)
Surgery with radiotherapy	10 (11.0)	9 (14.3)
Chemoradiotherapy	37 (40.7)	32 (50.8)
Surgery with chemoradiotherapy	7 (7.7)	6 (9.5)
Surgery	5 (5.5)	–
Chemotherapy	1 (1.1)	–
Others (loss of follow-up before starting treatment)	7 (7.7)	–
**Smoking**		
No	14 (15.4)	11 (17.4)
Yes	76 (83.6)	52 (82.6)
Unknown	1 (1.0)	0 (0)
**Alcohol consumption**		
No	12 (13.2)	8 (12.7)
Yes	78 (85.8)	55 (87.3)
Unknown	1 (1.0)	0 (0)
**Nutritional diagnosis (PG-SGA)**		
Well nourished	34 (37.4)	27 (42.9)
Suspected malnutrition or moderately malnourished	44 (48.4)	30 (47.6)
Severe malnutrition	13 (14.3)	6 (9.6)
**BMI,** mean (SD)	22.89 (4.6)	23.4 (4.7)

*Abbreviations*: *SD* Standard Deviation, *BMI* Body mass index, *PG-SGA* Patient-Generated Subjective Global Assessment

The duration of antineoplastic treatment was approximately eight weeks, with daily sessions of radiotherapy, from Monday to Friday, totaling 38 to 40 sessions. According to the institution protocol, patients underwent radiotherapy with a total final radiation dose of 70 Gy or 72 Gy, with daily doses of 180 cGy or 200 cGy. The mean ± standard deviation of sessions of radiotherapy performed by the study patients was 1.40 ± 2.09 in T0, 20.00 ± 3.91 in T1 and 35.68 ± 4.84 in T2. For patients undergoing chemotherapy, the protocol consisted of weekly sessions of intravenous cisplatin at a dose of 40 mg/m^2^ over a period of seven weeks.

**Fig. 2:**
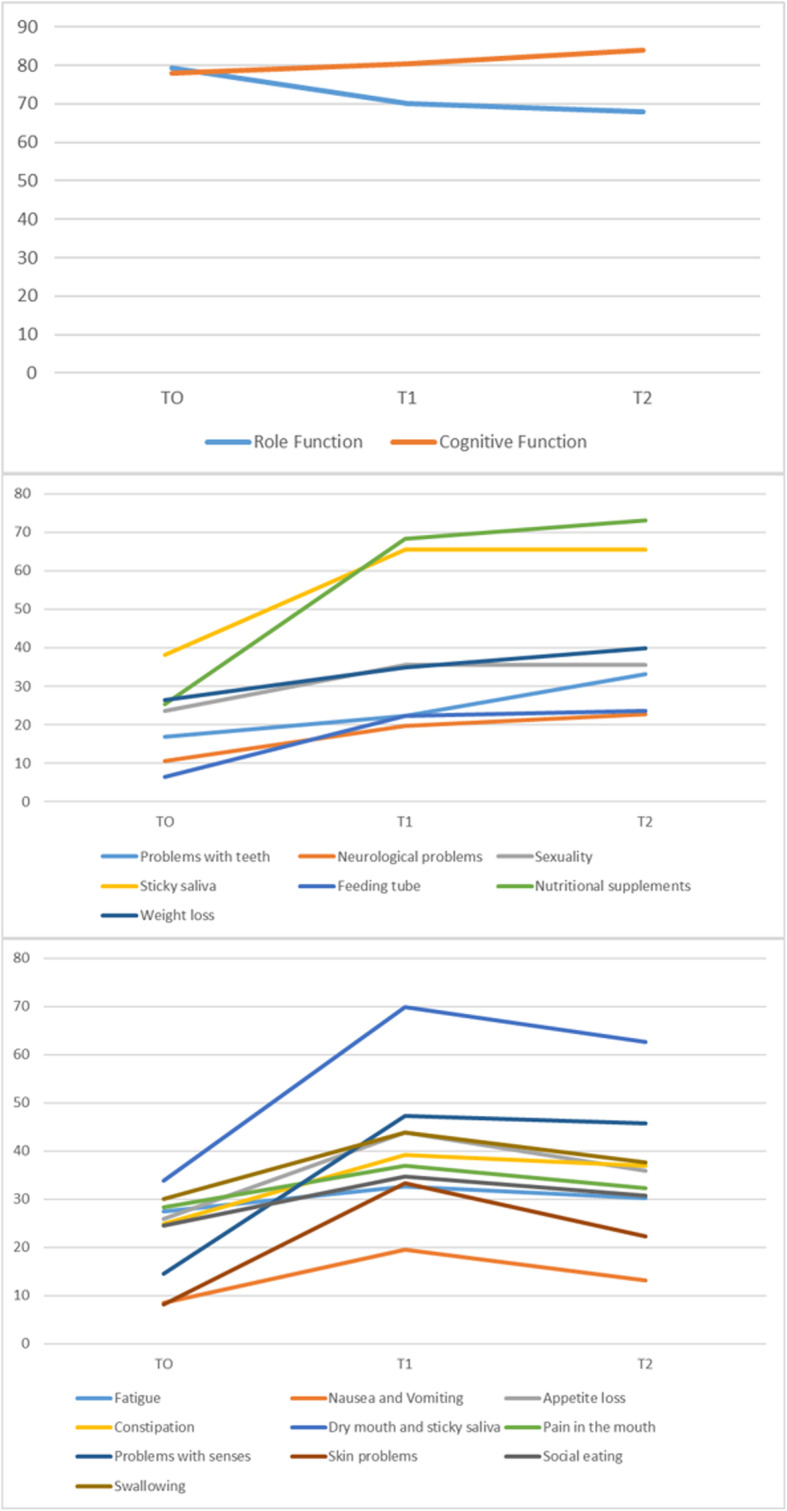
Alteration of the mean score during the different study phases: Funcional scales, single items and symptom scales which presented statistically significant difference by Friedman’s non-parametric test (T0: before or at beginning; T1: the middle of treatment T2: immediately at the end of treatment). The data are presented in the following order: functional scales, symptoms scales and items that got worse at T1 and kept getting worse at T2 and scales, symptoms and items that got worse at T1 and showed a slight improvement at T2

Regarding HRQOL, no differences were found between the global health status at different time points (Table 2). Conversely, QoL became worse (shown by decreasing scores) in ‘role’ functional scale comparing T0-T1 and T0-T2 (*p* = 0.009). An improvement in scores during treatment was observed for the domain of cognitive function (*p* = 0.035); (Fig. 2).

**Table 2:** QLQ-C30:Scores of Quality of life of patients with head and neck cancer during antineoplastic treatment (n = 63)

Quality of Life domains	T0		T1		T2		***p-value***	∆ T1-T0 n (%)	∆ T2-T1 n (%)	∆ T2-T0 n (%)
	Mean (SD)	Median (p_**25**_-p_**75**_)	Mean (SD)	Median (p_**25**_-p_**75**_)	Mean (SD)	Median (p_**25**_-p_**75**_)		≥ ± 10 score points n (%)		
Global health status	67.46 (20.61)	66.66 (50.00–83.33)	69.31 (21.62)	66.66 (50.00–83.33)	61.12 (21.40)	66.66 (50.00–83.33)	0.622	13 (20.6)	20 (31.7)	18 (21.6)
Physical function	80.39 (19.26)	90 (65–100)	81.19 (18.24)	85 (70–100)	79.12 (18.82)	80 (60–100)	0.909	16 (25.4)	18 (28.6)	22 (34.9)
Role function	79.36 (30.92)	100 (66.66–100)	70.10 (35.05)	83.33 (50–100)	67.98 (32.83)	66.66 (50–100)	**0.009**	23 (36.5)	21 (33.3)	29 (46.0)
Emotional function	68.12 (29.31)	75 (41.66–91.66)	69.84 (29.19)	75 (50–100)	69.70 (28.60)	75 (50–100)	0.986	19 (30.2)	18 (28.6)	19 (30.2)
Cognitive function	78.04 (29.45)	100 (50–100)	80.42 (27.19)	100 (66.66–100)	83.86 (27.10)	100 (83.33–100)	**0.035**	12 (19.0)	11 (17.5)	8 (12.7)
Social function	85.44 (24.40)	100 (66.66–100)	80.95 (29.00)	100 (66.66–100)	81.48 (29.65)	100 (66.66–100)	0.619	17 (27.0)	11 (17.5)	17 (27.0)
Fatigue	27.51 (31.03)	11.11 (0.0–44.44)	32.62 (29.65)	22.22 (11.11–55.55)	30.15 (28.46)	22.22 (0.0–44.44)	**0.010**^**a**^	30 (47.6)	28 (44.4)	33 (52.4)
Nausea and vomiting	8.46 (20.71)	0.0 (0.0–0.0)	19.57 (24.04)	16.66 (0.0–33.33)	13.22 (25.07)	0.0 (0.0–16.66)	**< 0.001**^**b**^	30 (47.6)	9 (14.3)	15 (23.8)
Pain	32.27 (33.84)	16.66 (0.0–50)	30.68 (28.74)	33 (0.0–50)	27.77 (31.82)	16.66 (0.0–50)	0.799	23 (36.5)	20 (31.7)	22 (34.9)
Dyspnoea	11.11 (26.77)	0.0 (0.0–0.0)	13.22 (29.04)	0.0 (0.0–0.0)	13.22 (27.78)	0.0 (0.0–0.0)	0.821	8 (12.7)	8 (12.7)	9 (14.3)
Insomnia	29.10 (39.02)	0.0 (0.0–66.66)	22.22 (35.92)	0.0 (0.0–33.33)	25.39 (37.25)	0.0 (0.0–66.66)	0.630	10 (15.9)	13 (20.6)	11 (17.5)
Appetite loss	25.92 (36.63)	0.0 (0.0–66.66)	43.91 (44.32)	33.33 (0.0–100)	35.97 (43.29)	0.0 (0.0–100)	**0.015**	28 (44.4)	12 (19.0)	23 (36.5)
Constipation	24.86 (38.31)	0.0 (0.0–33.33)	39.15 (41.71)	33.33 (0.0–100)	37.03 (41.95)	33.33 (0.0–66.66)	**0.023**	24 (38.1)	18 (28.6)	21 (33.3)
Diarrhea	1.05 (5.89)	0.0 (0.0–0.0)	6.34 (20.61)	0.0 (0.0–0.0)	6.34 (23.07)	0.0 (0.0–0.0)	0.190	7 (11.1)	5 (7.9)	6 (9.5)
Financial difficulties	34.39 (41.02)	0.0 (0.0–66.66)	34.92 (42.09)	0.0 (0.0–100)	39.68 (43.93)	33.33 (0.0–100)	0.744	14 (22.2)	14 (22.2)	19 (30.2)

*P*-values followed by letters differed statistically according to the post hoc test at the 5% probability level (Friedman test and Dunn post hoc test ajusted by Bonferroni: ^a^ difference between T0 and T2; ^b^ difference between T0 and T1. ∆ T1-T0, ∆ T2-T1 and ∆ T2-T0 ≥ 10 or ≤ −10 score points: clinically significant negative difference

Symptoms scales also got worse (shown by an increase in scores) for fatigue (*p* = 0.010), nausea and vomiting (p = < 0.001) and appetite loss (*p* = 0.015) during the interval between T0 and T1, with either maintenance or discrete reduction between T1 and T2. This indicated a worsening in QoL in the middle of treatment (T1) which was maintained until the end of treatment (T2), but always with worse values as compared to T0 (Table 2). We observed same results for scores of dry mouth and sticky saliva, pain in the mouth, neurological problems, sexuality, problems with senses, skin problems, social eating, swallowing, weight loss, oral nutritional supplements and feeding tube (Tables 3 and 4, Fig. 2).

**Table 3:** QLQ-H&N43: Quality of life of patients with head and neck cancer during antineoplastic treatment (n = 63)

Quality of Life domains	T0		T1		T2		***p-value***	∆T1-T0 n (%)	∆T2-T1 n (%)	∆T2-T0 n (%)
	Mean (SD)	Median (p25-p75)	Mean (SD)	Median (p25-p75)	Mean (SD)	Median (p25-p75)		≥ ± 10 score points n (%)		
Anxiety	38.88 (38.21)	33.33 (0.00–66.66)	35.44 (34.45)	33.33 (0.00–66.66)	32.01 (29.82)	33.33 (0.00–50)	0.662	20 (31.7)	19 (30.2)	21 (33.3)
Body image	19.40 (28.70)	0.0 (0.0–33.33)	19.92 (25.49)	11.11 (0.0–33.33)	24.51 (29.54)	11.11 (0.0–44.44)	0.382	22 (34.9)	21 (33.3)	24 (38.1)
Coughing	30.15 (37.72)	0.0 (0.00–66.66)	33.33 (36.41)	33.33 (0.00–66.66)	34.92 (38.06)	33.33 (0.00–66.66)	0.680	18 (28.6)	21 (33.3)	21 (33.3)
Dry mouth and sticky saliva	33.86 (30.07)	33.33 (0.0–50)	69.84 (29.19)	66.66 (33.33–100)	62.69 (30.48)	66.66 (33.33–100)	**< 0.001**^**c**^	43 (68.3)	17 (27.0)	40 (63.3)
Lymphoedema	25.39 (40.03)	0.0 (0.0–33.33)	17.98 (32.69)	0.0 (0.0–33.33)	15.34 (29.22)	0.0 (0.0–33.33)	0.059	8 (12.7)	8 (12.7)	10 (15.9)
Neurological problems	10.58 (24.55)	0.0 (0.0–0.0)	19.57 (33.13)	0.0 (0.0–33.33)	22.75 (34.81)	0.0 (0.0–33.33)	**0.001**	15 (23.8)	10 (15.9)	17 (27.0)
Trismus or Opening mouth	18.51 (36.30)	0.0 (0.0–0.0)	22.22 (36.41)	0.0 (0.0–33.33)	28.57 (39.19)	0.0 (0.00–66.66)	0.113	10 (15.9)	13 (20.6)	14 (22.2)
Pain in the mouth	28.30 (29.99)	16.66 (0.0–41.66)	36.90 (29.43)	33.33 (8.33–58.33)	32.27 (25.68)	25 (8.33–50)	**0.012**^**b**^	29 (46.0)	12 (19.0)	28 (44.4)
Sexuality	23.54 (35.24)	0.0 (0.0–50)	35.44 (40.87)	0.0 (0.0–66.66)	35.44 (41.63)	0.0 (0.0–66.66)	**0.020**	26 (41.3)	13 (20.6)	23 (36.5)
Social contact	20.63 (37.59)	0.0 (0.0–33.33)	23.80 (38.99)	0.0 (0.0–66.66)	24.33 (36.02)	0.0 (0.0–66.66)	0.613	12 (19.0)	13 (20.6)	13 (20.6)
Problems with senses	14.55 (24.03)	0.0 (0.0–16.66)	47.35 (33.49)	50 (16.66–66.66)	45.76 (30.37)	50 (16.66–50)	**< 0.001**^**c**^	45 (71.4)	18 (28.6)	47 (74.6)
Shoulder Problems	12.16 (22.83)	0.0 (0.0–16.66)	10.31 (21.46)	0.0 (0.0–0.0)	10.58 (23.81)	0.0 (0.0–0.0)	0.627	7 (11.1)	12 (19.0)	11 (17.5)
Skin problems	8.11 (12.33)	0.0 (0.0–11.11)	31.39 (23.74)	33.33 (11.11–44.44)	26.63 (21.07)	22.22 (11.11–44.44)	**< 0.001**^**c**^	47 (74.6)	23 (36.5)	41 (65.1)
Social eating	24.60 (31.12)	8.33 (0.0–41.66)	34.65 (27.19)	25 (16.66–50)	30.68 (26.59)	25 (8.33–50)	**0.009**^**b**^	34 (54.0)	14 (22.2)	29 (46.0)
Speech	31.85 (31.59)	20 (6.66–60)	31.21 (33.58)	20 (0.0–60)	33.12 (32.17)	20 (6.66–60)	0.729	15 (23.8)	20 (31.7)	18 (28.6)
Swallowing	30.02 (30.06)	25 (0.0–58.33)	43.78 (30.63)	41.66 (16.66–75.00)	37.56 (29.70)	33.33 (8.33–58.33)	**0.014**^**b**^	31 (49.2)	12 (19.0)	28 (44.4)
Problems with teeth	16.75 (23.51)	0.0 (0.0–33.33)	22.22 (25.32)	11.11 (0.0–33.33)	33.15 (68.12)	33.33 (0.0–33.33)	**0.002**^**a**^	25 (39.7)	22 (34.9)	33 (52.4)
Weight loss	26.45 (36.92)	0.0 (0.0–41.66)	34.92 (40.35)	0.0 (0.0–66.66)	39.78 (40.41)	33.33 (0.0–66.66)	**0.004**	21 (33.3)	15 (24.2)	26 (41.9)
Problems with wound healing	6.87 (23.30)	0.0 (0.0–0.0)	4.76 (16.78)	0.0 (0.0–0.0)	5.29 (17.15)	0.0 (0.0–0.0)	0.978	4 (6.3)	5 (7.9)	6 (9.5)

*P*-values followed by letters differed statistically according to the post hoc test at the 5% probability level (Friedman test and Dunn post hoc test ajusted by Bonferroni: ^a^ difference between T0 and T2; ^b^ difference between T0 and T1; ^c^ difference between T0 and T1, and T0 and T2. ∆ T1-T0, ∆ T2-T1 and ∆ T2-T0 ≥ 10 or ≤ −10 score points: clinically significant negative difference

**Table 4:** QLQ-H&N35:Quality of life of patients with head and neck cancer during antineoplastic treatment (n = 63)

Quality of Life domains	T0		T1		T2		***p-value***	∆T1-T0 n (%)	∆T2-T1 n (%)	∆T2-T0 n (%)
	Mean (SD)	Median (p25-p75)	Mean (SD)	Median (p25-p75)	Mean (SD)	Median (p25-p75)		≥ ± 10 score points n (%)		
Speech problems	27.33 (31.54)	11.11 (0.0–55.55)	29.10 (32.08)	33.33 (8.33–58.33)	32.27 (25.68)	25 (0.0–50)	0.139	23 (36.5)	27 (42.9)	26 (41.3)
Social contact	9.62 (14.55)	0.0 (0.0–13.33)	13.75 (20.13)	0.0 (0.0–20)	13.86 (18.67)	6.66 (0.0–20)	0.211	20 (31.7)	14 (22.2)	19 (30.2)
Teeth	11.64 (28.81)	0.0 (0.0–0.0)	14.81 (32.66)	0.0 (0.0–0.0)	13.75 (29.71)	0.0 (0.0–0.0)	0.753	9 (14.3)	8 (12.7)	10 (15.9)
Opening mouth or trismus	18.51 (36.30)	0.0 (0.0–0.0)	22.22 (36.41)	0.0 (0.0–33.33)	28.57 (39.19)	0.0 (0.00–66.66)	0.113	10 (15.9)	13 (20.6)	14 (22.2)
Dry mouth	18.51 (36.30)	0.0 (0.0–0.0)	22.22 (36.41)	0.0 (0.0–33.33)	28.57 (39.19)	0.0 (0.00–66.66)	0.113	10 (15.9)	13 (20.6)	14 (22.2)
Sticky saliva	38.09 (39.19)	33.33 (0.0–66.66)	65.60 (39.24)	66.66 (33.33–100)	65.60 (37.84)	66.66 (33.33–100)	**< 0.001**^**a**^	39 (61.9)	16 (25.4)	38 (60.3)
Felt ill	24.86 (37.84)	0.0 (0.0–66.66)	25.92 (37.12)	0.0 (0.0–33.33)	31.21 (39.65)	0.0 (0.00–66.66)	0.147	15 (23.8)	18 (28.6)	9 (14.3)
Pain killers	63.49 (48.53)	100 (0–100)	69.84 (46.26)	100 (0–100)	69.84 (46.26)	100 (0–100)	0.467	9 (14.3)	7 (11.1)	9 (14.3)
Nutritional supplements	25.39 (43.87)	0.0 (0–100)	68.25 (46.92)	100 (0–100)	73.01 (44.74)	100 (0–100)	**< 0.001**^**a**^	29 (46.0)	8 (12.7)	31 (49.2)
Feeding tube	6.34 (24.58)	0.0 (0.0–0.0)	22.22 (41.90)	0.0 (0.0–0.0)	23.60 (42.93)	0.0 (0.0–0.0)	**0.001**	10 (15.9)	4 (6.3)	12 (19.0)
Weight loss	50.79 (50.39)	100 (0.0–100)	53.96 (50.24)	100 (0.0–100)	52.38 (50.34)	100 (0.0–100)	0.924	16 (25.4)	9 (14.3)	14 (22.2)
Weight gain	25.39 (43.87)	0.0 (0.0–100)	31.74 (46.92)	0.0 (0.0–100)	42.85 (49.88)	0.0 (0.0–100)	0.076	14 (22.2)	16 (25.4)	17 (27.0)

*P*-values followed by letters differed statistically according to the post hoc test at the 5% probability level (Friedman test and Dunn post hoc test ajusted by Bonferroni: ^a^ difference between T0 and T1, and T0 and T2. ∆ T1-T0, ∆ T2-T1 and ∆ T2-T0 ≥ 10 or ≤ −10 score points: clinically significant negative difference

In the same way, a high frequency of clinically altered values (considering deltas by time) was observed in scores. The score for global health status, for example, was not different at the different time points. Nevertheless, we observed that 20% of patients had an important clinical change over time between T1-T0, 21.6% for T2-T0 and 31.7% for T2-T1 for this domain. The same could be observed for other domains that also showed clinically significant negative differences between times ranging from 6 to 74%. Dry mouth and stick saliva, nausea and vomiting, social eating and fatigue, for example, showed high clinical differences that must be taken into account.

## Discussion

Our results demonstrated a worsening of HRQOL in ‘role’ functional scale and symptoms scales, especially in the middle of treatment (T1), indicating an influence of therapy in short-term, within the first month after initiation, and that this worsening continues until the end of treatment (T2). Some domains were not associated with significant changes over time, such as global status score. However, when we analyzed the deltas at the different time points of study, we verified a significant portion had a worsening in QoL, about 6 to 74% of patients presented negative, clinically significant differences. Our study is the first one to evaluate the impact of treatment on QoL in short-term. Furthermore, we used the revised module EORTC QLQ-H&N43 and we have considered the clinical significance of QoL scores.

Many studies that evaluated HRQOL before and after treatment in HNC patients were also unable to find differences in health global status scores [22, 23], neither in a systematic review or meta-analysis, when comparing QoL pre-treatment and during follow-up [21]. It is likely that global health is complex and difficult for patients to measure. On several occasions it has been noted that this domain is abstract; patients have reported a gratitude for being alive and having the opportunity to be treated, maintaining a more optimistic view of life. So, their perception may not incorporate all the complexity of domain [24]. In addition, the global QoL is more sensitive to the so-called “response shift” characterized by an internal reframing of the patient in relation to his perception of health [11].

Regarding the functional scales, ‘role’ function showed greater differences in scores, but the emotional function scores were not different. Conversely, fatigue increased by 200% from T1 to T0 and T2 to T0. The increase in fatigue scores after treatment has also been found by other studies [2, 6], and this included the occurrence of symptoms at different moments of radiotherapy (before treatment, mid-treatment, at the end of treatment and 1 month after the end of treatment) [6].

A previous study found a positive association between fatigue and inflammation during treatment in patients with HNC, although the mechanisms have not yet been elucidated [25]. HNC patients undergoing chemoradiotherapy with cisplatin showed an increase in urinary excretion of carnitine, impairing energy metabolism and contributing towards fatigue [26]. Another factor that may contribute to fatigue in these patients is the presence of symptoms that could lead to reduced food intake [27, 28], such as the dry mouth and pain in the mouth or throat [16, 29, 30].

The domains of symptoms of nausea and vomiting, appetite loss, dry mouth and sticky saliva, pain in the mouth, swallowing, problems with teeth and social eating problems were associated with the time course of treatment. For all the domains, an increase in scores in the middle of treatment, and maintenance of this higher score until the end of treatment, was observed. Other studies also observed an increase in scores for symptoms with greatest impact on nutrition after treatment [14, 22], leading to a reduction in food intake and favoring the occurrence and/or worsening of nutritional status [31, 32]. On the other hand, previous studies found a positive association between nutritional status and QoL [16, 22], as expected.

A dry mouth is one of the main symptoms reported by HNC patients undergoing radiotherapy and is associated with damage of salivary glands, resulting in hyposalivation that leads to a feeling of dryness. This effect compromises taste, chewing and swallowing and favors oral infections, such as dental caries [33]. Swallowing disorders may also occur by fibrosis secondary to radiotherapy, and this modality of treatment can also lead to damage of taste buds, leading to dysgeusia [34]. This set of changes leads to nutritional deterioration of these patients, affecting their QoL.

In addition, these patients may require changes in consistency of food and oral supplementation, and sometimes need to have feeding tubes inserted [7, 34]. In our study, the patients needed greater oral nutritional supplementation in T1 (66.7%) and T2 (73%) as compared to T0 (30.2%), indicating an increase in the need to complement the diet as the treatment progressed.

The perceptions of patients in relation to QoL were observed in a previous study [7]. These patients reported some problems in using the feeding tube, including a feeling of shame caused by visible presence of tube and a feeling of missing eating and drinking orally. In contrast, the patients related some positive points, including nutritional comfort of not having to worry about not being able to swallow and not losing weight. However, despite this, these changes can lead to losses in social relationships associated with food.

In our results, we identified an increase in problems related to social feeding. Food problems can lead to considerable psychological and social problems [35, 36]. These patients are often presented as being unsafe for eating out, gain the least pleasure from their meals, and can find eating out stressful for extended period [35].

In the same way, the domains of constipation and other symptoms showed an increase in scores during treatment, indicating a worsening condition in these areas. Constipation in cancer patients is multifactorial and may be related to increased age, diet, reduced physical activity, psychological aspects, chemotherapeutic medications and opioids [37].

The sexuality of patients was also affected during treatment, with an increase of 150% in the median scores at times T1 and T2 as compared with T0. Individuals with HNC have a greater risk of problems with sexuality, mediated by the effects of the disease and treatment on body image, resection and mutilation, and functional and psychological problems [38]. Although we did not find a significant difference for body image scores, emotional problems or physical function, we observed clinically important changes in scores from the time point T0 to T1 and from T0 to T2, indicating a worsening in these domains during the long treatment. Corroborating with our findings, other studies also found a negative impact of treatment on the sexuality of these patients [2, 39].

Finally, neurological problems increased throughout treatment (T0-T1 and T0-T2). Neuropathic symptoms in HNC patients are multifactorial and may be related to brachial plexopathy for neck dissection, higher doses of radiation and chemoradiotherapy [40], especially with utilization of cisplatin [41, 42].

In general, HRQOL became worse in short-term, evidenced in the first month of treatment (mainly T0-T1 and T0-T2) in HNC patients. The scientific literature is broad in relation to studies reporting long-term QoL impairment in these patients; however, our results suggest an important short-term impact during treatment. This context is important to consider, because advances in treatment, increased survival and cases of HNC in younger individuals [38] lead to a greater need for adoption of actions around early prevention, counseling, management and support for these patients.

This study has some limitations. Firstly, the different treatment combinations of chemotherapy and radiotherapy were not considered in these analyses. Furthermore, the treatment completion rates and the times required to complete the prescribed courses of therapies were not analysed in details. These factors could impact differently on QoL in these patients. However, the combination of chemotherapy and radiotherapy was the most common treatment strategy. Secondly, there are some additional potential confounding factors that we did not adjusted because of the ineherent complexity of the sample, for example, tumor side, tumor stage, sex, income, among others. Since the design study is prospective, patients are compared to themselves through time. So the adjustments by the mentioned points are not indeed mandatory. Despite these limitations, this study is the first to evaluate QoL during treatment (short-term context) and consider the practical importance of clinical significance of delta scores. In addition, this is the first study to use the revised module for quality of life assessment in HNC (EORTC QLQ - H&N43) and, thus, could help other studies and allow future psychometric and clinical comparisons.

The establishment of protocols is essential for early counseling and management of symptoms of nutritional impact. These symptoms, as demonstrated in this study, have a direct impact on QoL of patients and, consequently, on the continuation and success of treatment. In this context, appropriate and individualized nutritional accompaniment is fundamental for improvement of clinical outcomes in HNC.

## Conclusions

HRQOL became worse in short-term after treatment in patients with HNC. These effects appeared within the first month after starting treatment (in the middle of the antineoplastic treatment) and remained until the end of therapy. Multi-professional actions aimed at minimizing impairments in QoL should be an obligatory part of the care routine, in order to minimize the symptoms and to make treatment less exhausting and traumatic process, and easier to complete.

## Data Availability

The datasets used and/or analysed during the current study are available from the corresponding author on reasonable request.

## Abbreviations

EORTC: European Organization for Research and Treatment of Cancer
QoL: Quality of life
HNC: Head and Neck Cancer
HRQOL: Health-Related Quality of Life
QLQ H&N: Quality of Life Questionnaire Head and Neck cancer Module
SD: Standard Deviation
TNM: Tumor Node Metastasis
UICC: International Union for Cancer Control
AJCC: American Joint Committee on Cancer
PG-SGA: Patient-Generated Subjective Global Assessment
